# Fecundity disorders in older women: declines in follicular development and endometrial receptivity

**DOI:** 10.1186/s12905-020-00979-7

**Published:** 2020-06-01

**Authors:** Li Wang, Shulan Lv, Wenjun Mao, E. Bai, Xiaofeng Yang

**Affiliations:** Department of Gynecology and Obstetrics, The First Affiliated Hospital of Xi’an Jiaotong University, Xi’an, Shaanxi China

**Keywords:** Older women, Fecundity, Endometrial receptivity, Implantation window, Ultrasonic parameters

## Abstract

**Background:**

Little research is available on follicular development and endometrial receptivity in older women. This study aimed to assess follicular development and endometrial receptivity, and to evaluate ultrasonic parameters in predicting endometrial receptivity during the implantation window in older women.

**Methods:**

For this prospective case-control study, 224 older women and 215 young women were recruited. The follicular development and endometrial thickness were monitored by transvaginal ultrasound. During the implantation window, the pulsatility index (PI) and resistance index (RI) of the uterine arteries and subendometrial region, endometrial volume, vascularization index (VI), flow index (FI) and vascularization flow index (VFI) were calculated between the two groups. The ultrasonic parameters were used to assess endometrial receptivity in older women.

**Results:**

The serum anti-Mullerian hormone (AMH) concentration and antral follicle count (AFC) were significantly lower in older women than in young women. The average diameter of the dominant follicle on days 14, 16, and 18 of the menstrual cycle were significantly smaller, and the subendometrial region RI on days 12, 14, 16, and 18 of the menstrual cycle were significantly higher in older women than in young women. The normal ovulation rate was significantly lower in older women than in young women. The subendometrial region RI was significantly higher, and the endometrial VI, FI, and VFI were significantly lower in older women compared with young women. The biochemical pregnancy rate, clinical pregnancy rate and ongoing pregnancy rate of older women were significantly lower than in young women. The best ultrasonic parameter for predicting endometrial receptivity during the implantation window in older women was VI (AUC =0.889, sensitivity 92.6% and specificity 85.4%).

**Conclusions:**

Older women present decreased serum AMH concentrations and AFC, defined as indicators of ovarian reserve function. Older women are characterized by decreased follicular development and endometrial receptivity, which may lead to fecundity disorders.

## Background

In recent years, later-age childbearing has become a trend in China, particularly as more women have steadily entered the workforce. In addition, with the implementation of the two-child policy in China, the number of older women (≥35 years) who desire pregnancy is gradually increasing [1]. However, fecundity declines significantly beginning at approximately 32 years of age and decreases more rapidly after 37 years of age because of a decrease in egg quality and a gradual increase in the circulating level of follicle-stimulating hormone (FSH) [2]. Therefore, many older women will face fecundity disorders, including decreased egg quality, pregnancy rate and live birth rate, as well as increased embryonic aneuploidy and abortion rate [3].

It is well known that fecundity declines in older women are associated with decreased ovarian function and oocyte quality [4]. However, little research is available on endometrial receptivity in older women. Endometrial receptivity plays an important role in pregnancy, and women diagnosed with infertility have been shown to be associated with reduced endometrial receptivity. At present, ultrasonic imaging and expression of related biochemical markers in endometrium have been used to assess endometrial receptivity [5]. Transvaginal ultrasound is the first-line method to observe follicular development and endometrial growth because of its non-invasive characteristics, and ultrasonic parameters, including endometrial thickness, classification and blood flow, have been confirmed to reflect endometrial receptivity [6, 7]. This study aimed to assess ovarian reserve function, follicular development and endometrial receptivity in older women and to evaluate the predictive value for endometrial receptivity of different ultrasonic parameters during the implantation window.

## Methods

### Study design and participants

This prospective case-control study was conducted in the First Affiliated Hospital of Xi’an Jiaotong University from January 2017 to April 2019. A total of 224 older women (≥ 35 years) and 215 women under the age of 35 who wanted to become pregnant were recruited. Women were included if they had regular menstrual cycles (28–32 days) and partners with normal semen [8]. Women were excluded if they had polycystic ovarian syndrome, endometriosis, pelvic acute or chronic inflammatory diseases, abnormal thyroid function, or underwent gynaecological surgery. Women were also excluded if they were diagnosed with infertility or underwent assisted reproductive technology (ART) treatment. Socio-demographic information such as age, body mass index (BMI), number of gravidity and deliveries, previous obstetrical history and menstrual cycle were obtained by a questionnaire. Sex hormone and anti-Mullerian hormone (AMH) concentrations were tested in the laboratory of our hospital. The baseline antral follicle count (AFC) was determined, which was defined as the total number of antral follicles (follicles measuring 2–10 mm) in both ovaries. This study was approved by the Ethics Committee of The First Affiliated Hospital of Medical College of Xi’an Jiaotong University. All participants gave written informed consent according to the procedures.

### Outcome measures

All ultrasound scans were performed by one operator using a Voluson E8 (GE Medical Systems, USA) to avoid interobserver variation. The operator was blind to the group of the patients. The results of the ultrasound assessment did not affect subsequent clinical management procedures. Starting from the 8th day of the menstrual cycle, the follicular development, endometrial thickness (ET), pulsatility index (PI) and resistance index (RI) of the uterine arteries and subendometrial region were monitored by transvaginal ultrasound, and then the above indicators were calculated every 2 days before ovulation. If the average diameter of dominant follicle achieved 18 mm, then intercourse was suggested. The ET, PI and RI of the uterine arteries and subendometrial region were detected 6 to 7 days after ovulation (implantation window). The ET was measured in the midsagittal plane in the fundus of the uterus (point of maximal thickness) from the echogenic interface at the junction of the endometrium and myometrium. The pulsatility index (PI) and resistance index (RI) of the uterine arteries were obtained from the ascending main branch of the uterine artery on the left and right sides of the cervix in a longitudinal plane. The average value of left and right uterine arteries was used for the final statistical analysis. The subendometrial region was defined as within 5 mm of the echogenic endometrial borders. The cursor of the Doppler was positioned to where the vessel with good color signals was identified on the screen. The manual mode of the virtual organ computer-aided analysis (VOCAL) contour editor was used to cover the entire 3D volume of the endometrium with a 15° rotation step. A total of 12 endometrial slices were obtained outlining the endometrium at the myoendometrial junction from fundus to internal os. The endometrial volume, vascularization index (VI), flow index (FI) and vascularization flow index (VFI) were calculated automatically [9]. All ultrasonic parameters were measured three times, and the average value was used for the final statistical analysis. Ovulation was confirmed by the following criteria: (a) The dominant follicle **≥**18 mm, followed by the disappearance or reduction in size of the dominant follicle by more than 5 mm. (b) Appearance of free fluid in the Pouch of Douglas. (c) Midluteal (6 to 7 days after ovulation) serum progesterone was ≥15 nmol/L measured by chemiluminescence methods [10]. Luteinized unruptured follicular syndrome (LUFS) is dominant follicle which fail to ovulate, undergo luteinization, and may become increasingly filled with blood. LUFS was defined as persistent existence or enlargement of follicles after maturation, thickening of follicle walls, and a strength echo in the point or grid shape or a cystic solid echo with great tension inside the follicle [11].

The biochemical pregnancy, clinical pregnancy and ongoing pregnancy were compared during this menstrual cycle. The human chorionic gonadotropin concentration ≥ 10 mIU/mL detected at 2 weeks after ovulation was identified as biochemical pregnancy. The gestational sac in the uterine cavity detected by transvaginal ultrasound was identified as clinical pregnancy. A fetus with heart beats at 12 weeks of pregnancy was identified as ongoing pregnancy [12].

The primary outcome included follicular development and ultrasonic parameters of endometrial receptivity. The secondary outcomes were the pregnancy rates and the predictive value of different ultrasonic parameters for endometrial receptivity in older women.

### Statistical analysis

The data in this study were statistically analyzed by SPSS version 16.0. The Kolmogorov–Smirnov test was performed to examine the normal distribution before statistical tests. The normally distributed continuous variables were presented as mean ± standard deviation and analyzed by Student’s *t* test. Non-normally distributed data were given as median (25, 75% quartile range) and were analyzed by the Mann–Whitney *U* test. Differences in dichotomous outcomes were analyzed by chi-square test. The receiver operating characteristic (ROC) curves was used to assess the predictive value of different indicators for endometrial receptivity. Statistical significance level was set at *p* < 0.05.

## Results

The data in Table 1 suggest that the serum AMH concentration and AFC were significantly lower in older women than in young women (*p* < 0.05), but the percentage of parity (≥1) was significantly higher in older women than in young women (*p* < 0.05). There was no significant difference when comparing other basic clinical data between the two groups (*p* > 0.05).

**Table 1:** Clinical data between older women and young women

Characteristics	Older women (*n* = 224)	Young women (*n* = 215)	*P* value ^a^
Age (years)	38.59 ± 6.37	27.43 ± 5.29	0.019
BMI (kg/m^2^)	21.46 ± 5.45	22.09 ± 4.87	0.658
Gravidity	3 (2–6)	3 (2–5)	0.507
Parity			0.034
0	68 (30.36%)	86 (40.00%)	
≥1	156 (69.64%)	129 (60.00%)	
Abortion	2 (2–4)	2 (1–3)	0.065
Live birth	1 (1–2)	1 (1–1)	0.129
Previous obstetrical history			
GDM	16 (10.26%) ^b^	10 (7.75%) ^b^	0.465
HDP	13 (8.33%) ^b^	11 (8.53%) ^b^	0.953
Menstrual cycle (days)	30.12 ± 5.53	31.45 ± 6.02	0.225
Basic concentrations			
FSH (mIU/mL)	7.80 ± 2.76	6.35 ± 2.01	0.367
LH (mIU/mL)	5.53 ± 1.12	5.48 ± 1.29	0.216
PRL (ng/mL)	10.14 ± 3.37	8.45 ± 2.48	0.103
E_2_ (pmol/L)	91.35 ± 25.63	102.47 ± 30.42	0.298
P (nmol/L)	1.09 ± 0.76	1.17 ± 0.90	0.342
T (nmol/L)	0.92 ± 0.41	0.86 ± 0.44	0.677
AMH (ng/mL)	1.02 ± 0.58	2.65 ± 0.99	0.019
AFC (number)	8.53 ± 2.21	15.67 ± 4.32	0.022

*GDM* gestational diabetes mellitus, *HDP* hypertensive disorders of pregnancy. *FSH* follicle stimulating hormone, *LH* luteinizing hormone, *PRL* prolactin, *E*_*2*_ estradiol, *P* progesterone, *T* testosterone

Values are presented as mean ± SD, median (quartile range) or number (percentage).

^a^*t*-test, Mann-Whitney*U* test or Chi-square test

^b^ Percentage are calculated according to parity (≥1)

**Fig. 1:**
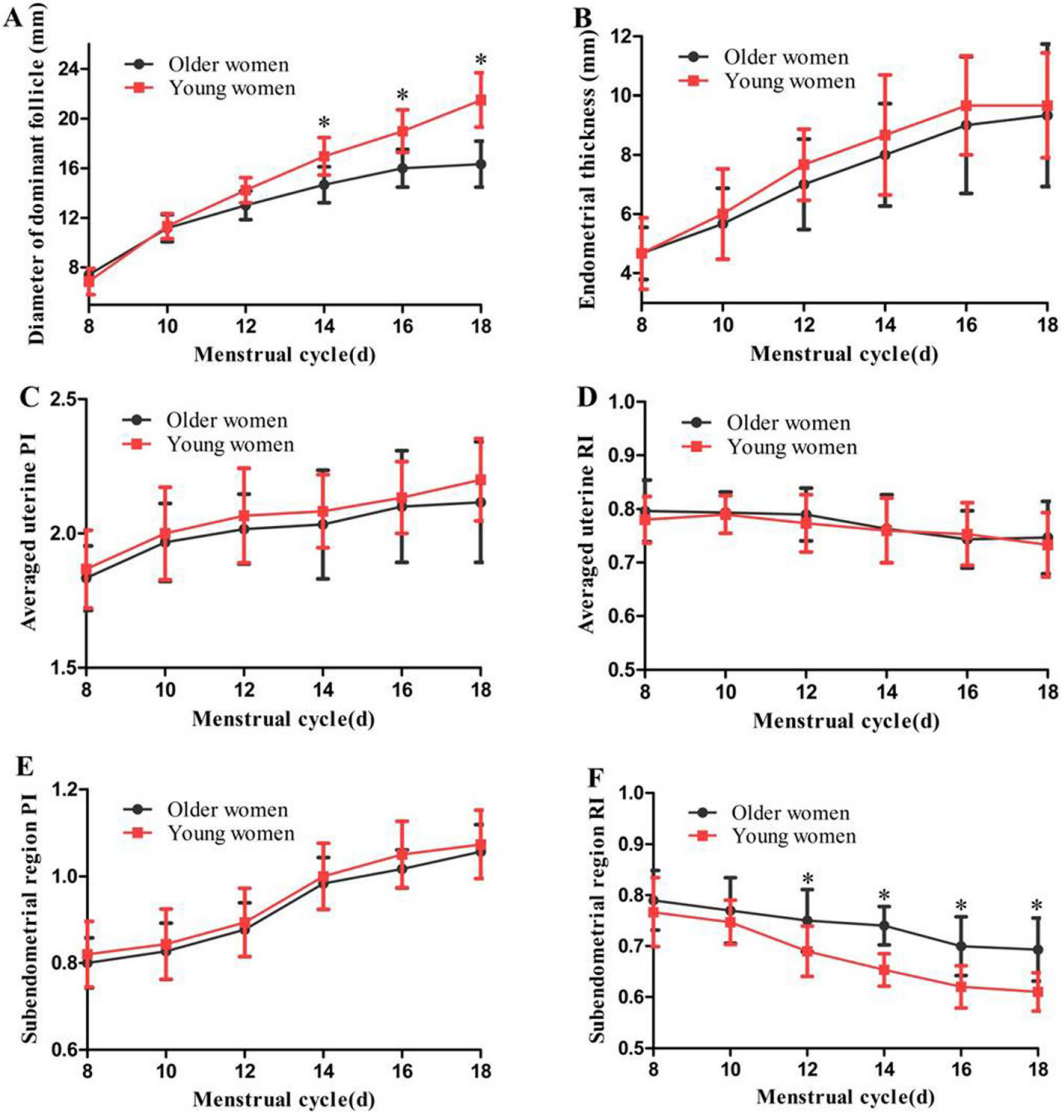
Follicular development, PI/RI of the uterine arteries and subendometrial region before ovulation between older women and young women (PI: pulsatility index, RI: resistance index, *p < 0.05). **a** and **b** Comparison of diameter of dominant follicle and endometrial thickness. **c** and **d** Comparison of averaged uterine PI and RI. **e** and **f** Comparison of subendometrial region PI and RI

Figure 1 indicates the follicular development and the PI/RI of the uterine arteries and subendometrial region before ovulation. The average diameter of the dominant follicle on days 14, 16, and 18 of the menstrual cycle were significantly smaller (*t* = 2.579, *p* = 0.041; *t* = 2.986, *p* = 0.032; *t* = 3.247, *p* = 0.025 respectively), and the subendometrial region RI on days 12, 14, 16, and 18 of the menstrual cycle were significantly higher in older women than in young women (*t* = 2.471, *p* = 0.046; *t* = 2.675, *p* = 0.039; *t* = 2.778, *p* = 0.036; *t* = 2.798, *p* = 0.035 respectively) (Fig. 1a and f). However, no significant difference was found when comparing endometrial thickness, average uterine PI and RI, and subendometrial region PI between the two groups (*p =* 0.346–0.995) (Fig. 1b–e).

**Fig. 2:**
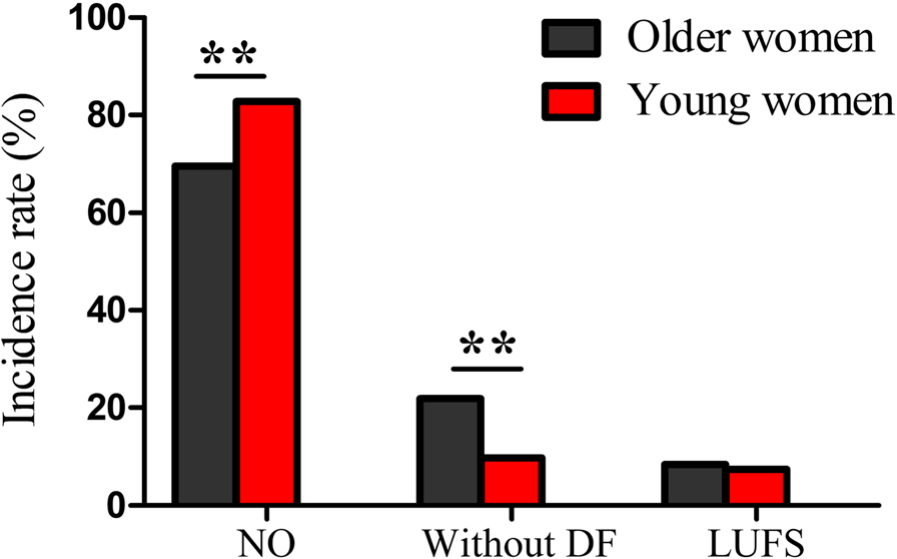
Follicular development and ovulation rate between older women and young women, ** *p* < 0.01 (NO: normal ovulation, DF: dominant follicle, LUFS: luteinized unruptured follicular syndrome)

Figure 2 reveals that the normal ovulation rate in older women was significantly lower compared with young women [69.64% (156/224) vs. 82.79% (178/215), *χ*^*2*^ = 10.421, *p =* 0.001], and the without dominant follicle rate in older women was significantly higher than that in young women [21.88% (49/224) vs. 9.77% (21/215), *χ*^*2*^ = 11.999, *p =* 0.001]. However, no significant difference was found when comparing the LUFS rate between the two groups [8.48% (19/224) vs. 7.44% (16/215), *χ*^*2*^ = 0.162, *p =* 0.687].

Table 2 shows the ultrasonic parameters of endometrial receptivity during the implantation window in women with normal ovulation between the two groups. The subendometrial region RI was significantly higher, and the endometrial VI, FI, and VFI were significantly lower in older women than in young women (*p* < 0.05). The data in Table 3 indicate that the biochemical pregnancy rate, clinical pregnancy rate and ongoing pregnancy rate of older women were significantly lower than those of young women (*p* < 0.05).

**Table 2:** Ultrasonic parameters of endometrial receptivity during implantation window in women with normal ovulation between the two groups

Parameters	Older women (*n* = 156)	Young women (*n* = 178)	*P* value ^a^
Uterine artery PI	2.03 ± 0.74	2.08 ± 0.81	0.897
Uterine artery RI	0.76 ± 0.13	0.75 ± 0.15	0.903
Subendometrial region PI	0.98 ± 0.24	1.00 ± 0.29	0.890
Subendometrial region RI	0.76 ± 0.12	0.62 ± 0.11	0.041
Endometrial thickness (mm)	9.01 ± 2.41	9.67 ± 2.68	0.335
Endometrial volume (cm^3^)	2.93 ± 0.87	3.24 ± 1.02	0.126
Endometrial VI (%)	1.72 ± 0.92	3.04 ± 1.13	0.019
Endometrial FI (0–100)	19.45 ± 4.65	32.3 ± 6.32	0.015
Endometrial VFI (0–100)	0.33 ± 0.02	0.97 ± 0.06	0.007

Values are presented as mean ± SD

^a^*t*-test

**Table 3:** Pregnancy rates between older women and young women

Parameters	Older women (*n* = 224)	Young women (n = 215)	*P* value ^a^
Biochemical pregnancy	22 (9.82%)	39 (18.14%)	0.012
Clinical pregnancy	17 (7.59%)	36 (16.74%)	0.003
Ongoing pregnancy	13 (5.80%)	34 (15.81%)	0.001

Values are presented as number (percentage).

^a^ Chi-square test

**Fig. 3:**
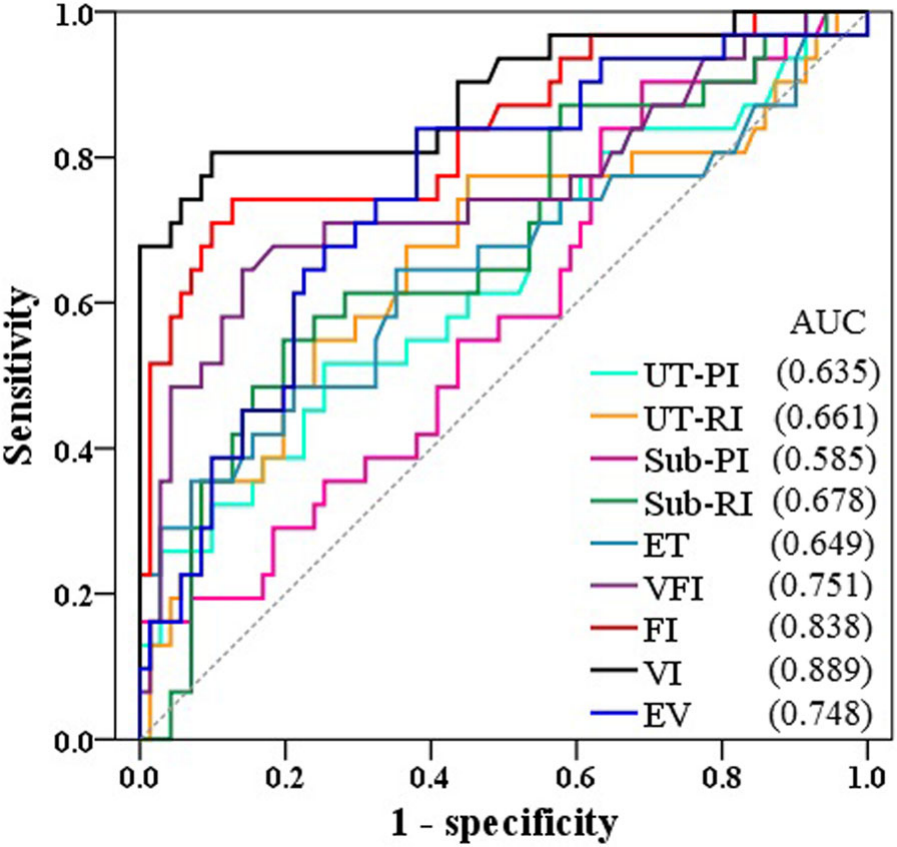
ROC curves of the predictive values for endometrial receptivity during implantation window in older women (UT-PI: uterine artery pulsatility index, UT-RI: uterine artery resistance index, Sub-PI: subendometrial region pulsatility index, Sub-RI: subendometrial region resistance index, ET: endometrial thickness, EV: endometrial volume, VI: vascularization index, FI: Flow index, VFI: vascularization flow index)

The predictive values of different ultrasonic parameters for endometrial receptivity in older women were displayed in Fig. 3. Data shows that VI has the highest predictive value (AUC = 0.889, sensitivity 92.6% and specificity 85.4%), followed by FI (AUC = 0.838, sensitivity 90.7% and specificity 82.1%).

## Discussion

Although there are several indicators to evaluate ovarian function, age is the primary determinant of reproductive potential [13]. Data from our study showed that the average diameter of the dominant follicle in older women was significantly smaller, and the normal ovulation rate was significantly lower in older women, which is associated with the fecundity declines of older women. Previous data suggested that among populations that do not use contraception, fertility rates decrease with increasing age of women [14]. The cumulative pregnancy rate observed across up to 12 insemination cycles was 74% for women younger than 31 years, decreased to 62% for women aged 31–35 years and decreased further to 54% for women older than 35 years [2]. Therefore, the pregnancy success rate of older women is significantly reduced in regard to both natural conception and assisted reproductive technology [15, 16]. Several indicators can be used to assess ovarian reserve function, including FSH, inhibin B, AMH and AFC. The data in this study revealed that AMH and AFC were superior to menstrual cycles and FSH in evaluating ovarian function in older women, which is consistent with the results reported in most previous research [17, 18]. As a woman ages, her oocyte and follicular pool declines, so AFC is a good indicator of ovarian function in women. As the oocyte and follicular pool declines, granulosa cells secrete less. Although the ability of AMH to predict reproductive potential is controversial, it is an excellent predictor of oocyte yield among women with infertility undergoing controlled ovarian hyperstimulation for in vitro fertilization (IVF) [19].

Previous studies have confirmed that most ovulation occurs within 48 h after the dominant follicle reached ≥18 mm in diameter. It has been reported that among healthy women trying to conceive, 30.7% pregnancies were initiated in a total of 625 natural menstrual cycles for which the dates of ovulation could be estimated. Conception occurred only when intercourse took place during a six-day period that ended on the estimated day of ovulation [20]. Data in our study showed that the biochemical pregnancy rates in young women and older women were 18.14 and 9.82%, which were lower than the above research results. This difference may be related to the existence of LUFS women in our study. In addition, our findings show that the biochemical pregnancy rate, clinical pregnancy rate and ongoing pregnancy rate in older women were significantly lower than those in young women. It has been shown that the clinical pregnancy rates were significantly lower in older women in standard IVF and ovum donation, and the decrease in endometrial receptivity with age was responsible for the higher rate of implantation failure in older women [21]. From a clinical perspective, extrapolating results obtained in young women with infertility to older is not justified. It is noteworthy that, if endometrial receptivity in older women is ignored by the clinician, the older women who were diagnosed with unexplained infertility may receive inappropriate therapies. These may expose women to unjustified risks and waste financial resources. Unfortunately, the available literature about follicular development and endometrial receptivity in older women during the natural menstrual cycle is limited and does not provide valid evidence. Therefore, randomized controlled trials aimed at identifying the follicular development and endometrial receptivity during the natural menstrual cycle in older women is needed.

The assessment of the endometrial receptivity has been a hotspot in the field of reproductive endocrinology, and its impairment has been confirmed to be associated with spontaneous abortion, infertility and repeated implantation failure [22, 23]. Embryo implantation is a complicated process in which the blastocyst interacts with the receptive endometrium. In the normal reproductive cycle of humans and mammals, there is a very short period during which the endometrium is receptive for embryo implantation. In the early stage of embryo implantation, angiogenesis is active, and the expression of various angiogenesis-related factors is increased, which provides support for embryonic development and pregnancy. Therefore, the blood supply of the endometrium is of great significance to its receptivity.

Early literature reported obvious changes occurred in endometrial volume and vascularization during the normal menstrual cycles, and three-dimensional energy Doppler ultrasound has been used to evaluate endometrial receptivity [24, 25]. The data in our study showed that the subendometrial region RI was significantly higher and that the endometrial VI, FI, and VFI during implantation window were significantly lower in older women than in young women, which might be related to the decreased endometrial receptivity. Furthermore, the data in this study showed that the best ultrasonic parameter for predicting endometrial receptivity in older women was VI, followed by FI. Our previous results showed that transvaginal two-dimensional ultrasound could evaluate endometrial receptivity by detecting endometrial thickness and blood flow [26]. Studies have confirmed that the value of this technique in evaluating endometrial receptivity is better than that of two-dimensional ultrasound, and it has been reported to be used to evaluate endometrial receptivity for in vitro fertilization-embryo transfer (IVF-ET) [27]. Wang et al. showed that increased endometrial blood flow in IVF-ET infertile women during follicular maturation was beneficial to pregnancy [28]. Other studies have found that three-dimensional energy Doppler ultrasonography can assess endometrial receptivity and predict pregnancy outcome by detecting follicular maturation day and embryo transfer day with intrauterine and subintimal blood flow [29].

The present study still has some limitations. Fecundity disorders in older women are associated with follicular dysplasia and increased aneuploidy. Furthermore, this was a prospective case-control study in a single centre, and the sample size was relatively small. Therefore, the repeatability of the results of this study needs to be confirmed by multi-centre surveys with large sample sizes.

## Conclusions

Older women present decreased ovarian reserve function, for which the predictive value of AMH and AFC is more sensitive. Older women present decreased follicular development and endometrial receptivity, which might be related to fecundity disorders. The results of this study provide new ideas for the improvement of pregnancy rate and reproductive outcomes in older women.

## Data Availability

The datasets used and/or analyzed during the current study are available from the corresponding authors on reasonable request.

## Abbreviations

BMI: Body mass index
AMH: Anti-Mullerian hormone
AFC: Antral follicle count
LUFS: Luteinized unruptured follicular syndrome
FSH: Follicle stimulating hormone
LH: Luteinizing hormone
PRL: Prolactin
E_2_: Estradiol
P: Progesterone
T: Testosterone
PI: Pulsatility index
RI: Resistance index
ET: Endometrial thickness
EV: Endometrial volume
VI: Vascularization index
FI: Flow index
VFI: Vascularization flow index
